# A public health research agenda informed by guidelines in development

**DOI:** 10.2471/BLT.17.200709

**Published:** 2017-12-01

**Authors:** Dermot Maher, Nathan Ford

**Affiliations:** a Special Programme on Research and Training in Tropical Diseases, World Health Organization, 20 avenue Appia, 1211 Geneva 27, Switzerland.; b Department of HIV and Global Hepatitis Programme, World Health Organization, Geneva, Switzerland.

The World Health Organization (WHO) is the leading and coordinating authority on public health within the United Nations system. Setting norms and standards, and shaping the research agenda are two of WHO’s six core activities.^1^ WHO can use this normative role to support the development of an agenda for public health research.

WHO develops global, clinical, programmatic and public health guidelines that support best practice in health delivery. In 2007, WHO established the Guidelines Review Committee to ensure that WHO produces high-quality guidelines that are based on internationally recognized methods and standards and are developed through a transparent, evidence-based decision-making process.^2^ Each guideline development process starts with the establishment of a guideline development group that includes leading experts in the field and relevant stakeholders from across all WHO Regions affected by the public health problem. The group may involve patients and those who most likely will implement the guidelines’ recommendations. The guideline development groups use systematic reviews of relevant evidence to make recommendations, and the Grading of Evidence, Assessment and Evaluation (GRADE) system to determine and qualify these recommendations.^3^ GRADE includes an appraisal of the quality of evidence and an assessment of potential benefits and harms, resource use, user values and preferences regarding the recommended intervention. The group considers these elements together to determine the direction and strength of a recommendation. When significant uncertainty exists with respect to the balance of an intervention’s benefits and harms, the guideline development group should describe the knowledge gap and set priorities for what further research is needed to address these gaps.^4^

Here we suggest that the WHO guideline development process be used as a foundation for building an agenda on public health research. We argue that this process provides a unique and efficient opportunity to compile an agenda from the research needs identified by each of the guideline development groups. Several aspects of the process support this suggestion. First, guideline development relies on comprehensive assessments of the evidence from high-quality systematic reviews, complemented with other sources of information. Second, identifying research gaps and needs is a core objective of any systematic review^5^ and a function of WHO guidelines.^6^ Third, leading experts review evidence from key systematic reviews to formulate recommendations. Fourth, the variety of stakeholders in the guidelines development group provides a much broader perspective for formulating research priorities than relying on academic researchers alone.

As one example, the guideline development group on postexposure prophylaxis for human immunodeficiency virus infection used GRADE as a starting point to formulate priority research questions, including describing the most feasible study designs and identifying potential biases.^7^ Twelve research questions were addressed using three study designs: (i) survey- and interview-driven research to identify barriers to access to postexposure prophylaxis and related clinical care; (ii) establishment of a global postexposure prophylaxis registry to generate data to inform the choice of an optimal drug regimen, record drug toxicities and track follow-up and linkage to care; and (iii) randomized controlled trials to determine the optimal strategies to promote adherence and successful outcomes.^7^ Positioning key clinical and programmatic research questions within a framework^4^^,^^8^ for assessing the strength of existing evidence can facilitate the formulation of an evidence-based research agenda and future revisions of guidelines.

However, the usefulness of the research priorities currently identified in each set of WHO guidelines is limited, because their quality, robustness, presentation and dissemination vary and WHO does not compile identified priorities on particular topics into a coherent agenda. Consequently, while a wide range of guideline development groups produce lists of research questions of variable detail on different topics, WHO is missing the opportunity to progressively provide a comprehensive agenda for public health research. A standardized approach to identifying research priorities through the guideline development process would not only feed into a WHO research agenda, but also likely encourage that more consistent attention is given to this step in guideline development.

An agenda for public health research would complement the work of the WHO Global Observatory on Health Research and Development on the priorities of health product research and development.^9^ In terms of identifying research priorities across the spectrum of health research, WHO would then cover two parts of the spectrum, product research and development, and public health. The development of an agenda for public health research would increase the public health impact of WHO guideline development; it would also influence and support governments and other bodies that support research.
